# The Last Mile in Beta-Cell Replacement Therapy for Type 1 Diabetes: Time to Grow Up

**DOI:** 10.3389/ti.2025.14565

**Published:** 2025-04-01

**Authors:** Lorenzo Piemonti

**Affiliations:** ^1^ Unit of Regenerative Medicine and Organ Transplants, IRCCS Ospedale San Raffaele, Milan, Italy; ^2^ Università Vita-Salute San Raffaele, Milan, Italy

**Keywords:** diabetes type 1, beta cell replacement therapy, islet, scalability, stem cell derived beta cells

## Abstract

Beta cell replacement therapy for type 1 diabetes (T1D) is undergoing a transformative shift, driven by advances in stem cell biology, gene editing, and tissue engineering. While islet transplantation has demonstrated proof-of-concept success in restoring endogenous insulin production, its clinical impact remains limited by donor scarcity, immune rejection, and procedural complexities. The emergence of stem cell-derived beta-like cells represents a paradigm shift, with initial clinical trials showing promising insulin secretion *in vivo*. However, translating these breakthroughs into scalable, widely accessible treatments poses significant challenges. Drawing parallels to space exploration, this paper argues that while scientific feasibility has been demonstrated, true accessibility remains elusive. Without a strategic shift, beta cell therapy risks becoming an elite intervention, restricted by cost and infrastructure. Lessons from gene and cell therapies for rare diseases highlight the dangers of unsustainable pricing and limited market viability. To bridge the “last mile” a Quality by Design approach is proposed, emphasizing scalability, ease of use, and economic feasibility from the outset. By emphasizing practical implementation over academic achievements, corporate interests, market economics, or patent constraints, beta cell therapy can progress from proof-of-concept to a viable, widely accessible treatment.

At the 48th Annual Conference ISPAD, held on 13–16 October 2022, in Abu Dhabi, UAE, I was invited to deliver a lecture titled *The Last Mile for Type 1 Diabetes cure* [1]. The intention behind this title was to highlight the ambivalence of the concept: while many interpreted it optimistically as signaling the imminent arrival of a definitive cure, the phrase also carries a cautionary meaning. In many fields, the “last mile” is often the most complex and challenging stage of development, requiring careful navigation to ensure successful implementation [2]. History teaches us that assuming victory just before the finish line is a surefire way to trip over our own shoelaces.

The field of beta cell replacement therapy for type 1 diabetes (T1D) is currently undergoing a remarkable transformation [3]. Over the past two decades, the well-established islet transplantation paradigm has provided compelling proof-of-concept evidence that restoring endogenous insulin production can lead to long-term glycemic control, protection from severe hypoglycemia, and improved quality of life [4–11]. However, the approach remains fundamentally constrained by the limited availability of organ donors, the need for lifelong immunosuppression, and the challenges associated with islet engraftment and survival [11–13]. In other words, we have a therapy that works beautifully—just for not enough people to make a real difference.

Recent breakthroughs in stem cell biology, tissue engineering, and gene editing are now reshaping the landscape, with the potential to overcome these intrinsic limitations [14–17]. The successful differentiation of stem cell-derived beta-like cells—whether from human embryonic stem cells (ESCs) or induced pluripotent stem cells (iPSCs)—into insulin-producing cells suitable for transplantation represents a paradigm shift [18–23]. Initial clinical trials have demonstrated the feasibility of this approach, with promising preliminary data showing functional insulin secretion *in vivo* [24–30]. The possibility of encapsulating or genetically engineering these cells to evade immune rejection could eventually obviate the need for chronic immunosuppression, further expanding the therapeutic potential [3, 4]. This progress is the culmination of decades of interdisciplinary research, bringing us to an exciting and optimistic phase in the field. But before we start popping champagne, let’s remember that many promising scientific advances have met their demise at the hands of real-world implementation challenges. As we celebrate these successes, it’s important to recognize the complexity of what lies ahead. While the idea of the “last mile” in beta-cell replacement may suggest we are nearing a definitive solution, history shows that the final phase often brings its most rewarding challenges, offering opportunities for further breakthroughs and innovation.

To illustrate this, we can draw an analogy to space exploration. Sending the first humans to the moon was one of the most significant technological feats in modern history, showcasing the scientific ingenuity of our species. Can we say that humanity has mastered lunar travel? Certainly. Have we transitioned from exploration to colonization? Not even close. The Apollo program, which successfully landed twelve men on the moon over 12 years, cost an estimated $288 billion in today’s currency and required the effort of 400,000 people. Was it worth it? Undoubtedly. The benefits of space exploration extended far beyond the moon landings themselves, driving innovations in computing, materials science, and medicine. But if our goal had been to establish a thriving lunar metropolis, we would have been woefully unprepared. The engineering required to sustain a permanent presence on the moon is vastly different from what was needed for brief exploration missions.

Similarly, while we have demonstrated that stem cell-derived beta cells can function in human recipients, scaling this intervention to treat millions of individuals with T1D presents a new set of challenges [31–34]. We’ve planted the flag, but we’re nowhere near ready to move in. For now, we must acknowledge that only a select few will have access to this groundbreaking therapy in its early stages. Let’s be realistic: sending twelve men to the moon was a tremendous achievement, but building the infrastructure to support thousands is an entirely different level of challenge—and a much greater one when scaling up to millions. If we continue to approach beta cell replacement with an “Apollo mission” mindset, we risk creating a therapy that could be limited in accessibility. This would necessitate either stringent stratification based on risk-benefit analysis or, in a more troubling scenario, allocation based on financial capacity [35, 36].

It’s important to note that the biomedical field, while it shares some characteristics with space exploration in terms of complexity, is inherently different. The decentralized, iterative nature of biomedical research allows for faster and more varied innovation, often driven by global collaboration, and offers a more dynamic landscape than the singular focus of space exploration. In this regard, the biomedical field has some distinct advantages, such as flexibility and the potential for rapid progress due to the contributions of many smaller, specialized teams rather than relying on a monolithic, top-down approach. But there are also disadvantages to this fragmented approach. Without a central focus, there is a risk of research becoming too diffuse, lacking the critical mass of knowledge and resources needed to make real breakthroughs in a timely manner. The dispersed nature of the research may lead to silos of knowledge, and sometimes, these separate efforts can lack the cohesion necessary to propel the field forward efficiently. In the case of beta-cell replacement therapy, for instance, without a unified, coordinated strategy, progress may be delayed, and key challenges, such as creating scalable and affordable solutions, could remain unresolved.

Perhaps we do not fully consider the complexities of the “last mile” in scientific progress, where the challenges of scaling and ensuring widespread accessibility can be more intricate and demanding than the initial breakthroughs themselves. A recent reflection on human genome editing serves as a case in point [37]. It has been suggested that polygenic genome editing could become feasible within the next three decades, with theoretical models indicating that it could significantly reduce susceptibility to diseases such as coronary artery disease, Alzheimer’s, depression, diabetes, and schizophrenia. This is an intriguing prospect with profound ethical implications, but one thing is already clear and underestimated: this approach is unlikely to be applied to a significant portion of the population within any realistic timeframe. Why? Because it would require *in vitro* fertilization for every individual undergoing genome editing [38]. Once again, we have explored the possibility, but we have not “colonized” it. For a more immediate comparison to beta cell therapy, let’s assume for a moment that, starting today, we could transplant pancreatic islets without requiring immunosuppression. Would that mean we have reached the last mile in curing type 1 diabetes? Not at all. The number of donors would remain severely limited, and the procedure, still highly dependent on skilled operators, could not be automated or broadly implemented. Therefore, only a small group would have access, and this does not even take into account the cost factor.

Indeed, when considering the “last mile” in cell-based therapies, one of the major challenges is the cost, which may prevent the therapy from being automated or widely implemented. The case of gene and cell therapies for rare diseases serves as a cautionary tale [39]. Several promising therapies have been approved but later withdrawn from the market due to unsustainable pricing models and difficulties in reimbursement [40, 41]. Notable examples include Glybera, the first gene therapy approved in Europe, which was withdrawn in 2017, after being deemed commercially unviable, and Strimvelis, a gene therapy for ADA-SCID, which faced similar market challenges. News has recently emerged about the suspension of the development and commercialization of the hemophilia B gene therapy fidanacogene elaparvovec (marketed as Beqvez in the United States and Durveqtix in Europe) by Pfizer [42]. Fidanacogene elaparvovec marks the ninth advanced therapy to be withdrawn from the European market since 2015, a significant figure considering that only 27 such therapies have reached commercialization in total [43]. Notably, no patients appear to have received the therapy after its approval in the United States. Its price tag—$3.5 million per patient—certainly does not lend itself to widespread adoption. Another shake-up in the sector came with the recent developments surrounding bluebird bio, Inc. Founded with the mission of developing gene therapies for rare diseases, the company had already sparked debate over the sustainability of advanced therapies back in 2021, following the simultaneous withdrawal of two gene therapies from the European market—one for beta-thalassemia and the other for cerebral adrenoleukodystrophy (the latter of which remained available for only 3 months). Once valued at $11 billion in 2018, bluebird bio faced mounting financial difficulties due to high development costs and limited market access. Recently, the company was acquired by U.S. funds for a mere $30 million—a staggering devaluation that underscores the economic challenges plaguing biotech firms specializing in advanced therapies.^1^ The model used for rare disease therapies may not be directly applicable to widespread conditions like T1D, especially when scaling therapies like beta-cell replacement. In rare diseases, high per-patient costs are manageable, but for large populations, cost-reduction strategies are essential. A key approach is leveraging economies of scale, particularly in allogeneic therapies, where a single batch can treat multiple patients, spreading fixed costs and reducing per-patient expenses. However, autologous therapies face limitations in this regard due to the need for individualized production, which results in higher costs. Advancements in automation and bioprocessing technologies could reduce costs for both autologous and allogeneic therapies, but there are risks. Despite technological innovations, therapies may remain financially unfeasible for large populations due to high raw material costs, specialized facilities, and regulatory hurdles. Additionally, cost-reduction efforts must not compromise the therapy’s quality or efficacy, as this could undermine its long-term success.

So, the real question is: how we can successfully and safely navigate the “last mile”? One option is to place unwavering faith in scientific progress, if what seems impossible today will inevitably become reality. After all, history is filled with once-fantastical ideas that have materialized into everyday technology. In *Star Trek* (1964), the crew communicated using sleek, flip-open devices—perfect for intergalactic adventures. Three decades later, Motorola’s StarTAC brought that vision to life. Waiting, as Samuel Beckett illustrated in *Waiting for Godot*, carries profound human dignity. But waiting can also turn into a tragicomedy if it assumes that progress has no intrinsic limits—that it is merely a matter of time.

A second option is to take a different approach, drawing inspiration from the Quality by Design (QbD) framework [44–46]. For those unfamiliar with it, QbD is, above all, a philosophy that shifts the focus from quality control to quality by intentional design. It emphasizes that quality should not be tested into a product but rather built into it from the very beginning. At the core of QbD is the Quality Target Product Profile (QTPP), which defines the desired characteristics of a product, guiding its entire development. Fundamentally, the QbD approach marks a shift from a reactive, retrospective evaluation to a proactive, predictive model. Traditional quality control methods often rely on detecting and correcting issues after production. In contrast, QbD anticipates critical points and constraints during the design phase, allowing for a better understanding of the boundaries within which the process must operate. This shift from “test-and-fix” to “design-and-predict” enables more robust, efficient, and scalable therapeutic solutions, particularly in emerging fields like cell therapy. This concept is particularly crucial in the field of cell therapy, where the QTPP is not just about the intrinsic properties of the cellular product itself. Unlike conventional pharmaceuticals, cell therapies are living drugs, meaning their effectiveness and behavior depend on the dynamic interaction with the patient receiving the treatment. This reciprocal relationship between the therapy and the individual means that the QTPP must account for factors such as patient-specific responses, variability in the cellular product, and the evolving nature of the treatment within the body.

It is not my intention here to delve into the numerous complex aspects associated with the QbD approach in the field of beta-cell transplantation, aspects that are far from irrelevant. For example, defining Critical Quality Attributes (CQAs) for stem cell-derived beta cells (such as insulin secretion kinetics and purity) requires standardized assays, which remain underdeveloped. Even the discussion regarding the quality and potency of human pancreatic islets remains extensive [47]. To provide a sense of this complexity, in the recent FDA discussions concerning the approval of the Biologics License Application for pancreatic islets,^2^ potency criteria were suggested based on parameters such as ≥70% viable islets, counting based on DTZ staining and microscopic evaluation, as well as the ratio of insulin secretion under high glucose stimulation to low glucose stimulation (≥1). Ironically, some probiotic strains, such as *Saccharomyces cerevisiae*, could potentially exhibit similar metrics under certain conditions [48], highlighting the complexity and challenges in defining appropriate potency criteria for beta cel replacement. Moreover, regulatory alignment with agencies like the FDA/EMA is also understated and warrants more attention. Regulatory agencies play a critical role in the successful development, approval, and commercialization of novel therapies. However, in the case of beta-cell replacement therapies, particularly those derived from stem cells, there is a need for more comprehensive alignment with regulatory standards and guidelines.

Instead, in this context, I propose the broader adoption of the QTPP concept. Rather than focusing solely on traditional product quality parameters, QTPP advocates for a more comprehensive, patient-centered approach—an approach especially vital in the realm of stem cell-based therapies like beta-cell transplantation. The challenge goes beyond simply creating a functional cellular product; it is also about ensuring its scalability, as a transformative therapy that remains accessible to only a few is little more than an academic achievement. Therefore, rather than concentrating exclusively on the intrinsic qualities of the cellular product, a key step should be the definition of the QTPP—not only in terms of what the therapy is, but also how it will be administered, how it will interact with the patient, and the broader context in which it will be applied (Figure 1). If this is the lens through which we view the problem, then as a physician-scientist, I must wonder: what kind of product would I actually want to use? Ideally, it would be cryopreserved and easily thawed at the bedside with warm water, compatible with a standard syringe, and administered much like a simple intramuscular injection—no operating room, no GMP facility for post-thaw reconstitution, no angiographic suite for infusion, etc… A final product of just a few milliliters, nothing more. From this endpoint, we must work backward, establishing constraints from the outset. The goal is not to design a product that functions beautifully under ideal conditions but one that remains viable when deployed at scale. The constraints should not reflect what is manageable for an expert in a specialized lab or clinic but what is operationally feasible for millions of patients worldwide. Returning to the Apollo 13 analogy, the lesson lies in NASA’s approach to solving the air filter crisis [49]. They could have designed the ideal filtration system from scratch—but instead, they worked within the limits of what was already onboard, using available materials to construct a viable solution. In today’s terms, this was an exercise in *design thinking*, and it is precisely the mindset we need before moving forward. Recognizing this early is critical. It informs the definition of QTPPs and *CQAs*, which, in turn, shape every aspect of development—including procedural simplicity, implant size, and invasiveness. Consider this: if we could eliminate the need for an angiographic suite, a surgical team, or anything beyond local anesthesia, would not that already be a breakthrough? Similarly, as a healthcare provider, I must consider: how much can I afford to spend on beta-cell therapy for an individual with T1D? Defining this is essential, as sustainability is a key element of scalability, and in my view, it should be incorporated into the QTPP definition. In this regard, there should be a stronger focus on academic research into the economics of Beta-Cell Replacement Therapy. Furthermore, it is crucial to recognize that the economic sustainability of therapies is not only about cost-effectiveness but also about broader financial considerations. This was demonstrated in the past with hepatitis C treatments in countries with advanced public welfare systems [50].

**FIGURE 1:**
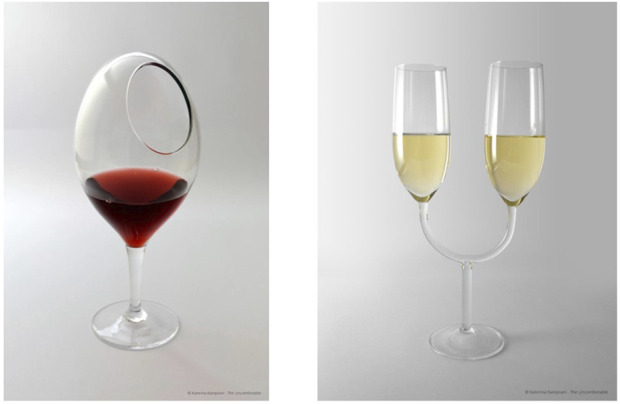
Two beautifully designed wine glasses that perfectly match the Quality Target Product Profile (QTPP) for a wine glass, yet fail in real-world usability due to a lack of consideration for the interaction with the drinker during the design and prediction phase. Both glasses exhibit ideal material quality, clarity, durability, and aesthetic appeal, fulfilling all standard QTPP criteria. They are made of high-quality, lead-free crystal, ensuring clarity and safety. Their shape and design feature an optimized bowl size and rim thickness to enhance aroma. The capacity and volume allow for proper aeration and optimal serving. They are scratch- and shatter-resistant, suitable for repeated use. Their weight and balance make them comfortable to hold, while their design ensures stability. They are easy to clean, dishwasher-safe, and resistant to stains and odors. Finally, they are scalable for mass production while maintaining quality. However, despite excelling in these technical attributes, the glasses overlook a crucial factor: the interaction between the glass and the drinker: they have an extravagant yet impractical design, making it impossible to drink from without spilling. This serves as a metaphor for the importance of a holistic approach in Quality by Design (QbD): a product must not only meet its defined quality criteria but also be practical, user-friendly, and functional in real-world applications—a principle that applies equally to wine glasses and therapeutic innovations. The represent glasees are part of “The Uncomfortable,” a collection of everyday objects that have been intentionally redesigned to be impractical by Athens-based architect Katerina Kamprani.

This phase should begin as soon as possible. Who should take responsibility for it? Undoubtedly, highly specialized academic centers with the necessary multidisciplinary expertise should lead the initial phase, overseeing both the design thinking process and, subsequently, the early-stage clinical trials to optimize the best conditions for implementation. These centers should work in close collaboration with the pharmaceutical industry, respecting each other’s areas of expertise and competencies. Following this initial development, a “hub-and-spoke” model should be adopted to enable broader dissemination. From these central expertise hubs, the process can gradually expand, ensuring that the therapy becomes accessible on a larger scale while maintaining the necessary standards of quality and feasibility. Supporting initiatives in this direction is essential, and projects like ACT (Accelerate Cell Therapies;^3^) by Breakthrough T1D serve as a prime example. ACT aims to significantly accelerate the availability of cell therapy products by uniting efforts in research, development, regulation, and clinical access. A core aspect of this initiative is the establishment of Clinical Centers of Reference for Cell Therapy—expert, multidisciplinary facilities that will play a key role in the fast-tracked adoption of “off-the-shelf” cell therapies. These centers will not only provide advanced treatments but also function as training hubs, helping other centers develop the expertise needed to deliver cutting-edge therapies. By prioritizing collaboration, training, and standardization of care, ACT and similar effort has the potential to ensure that life-changing therapies are accessible to millions of T1D patients worldwide.

A historical precedent for this approach can be found in bone marrow transplantation, which remains the only true cell therapy widely adopted on a global scale [51]. The foundations of this therapy were laid in the mid-20th century, with pioneering work by E. Donnall Thomas and George Mathé, who demonstrated that hematopoietic stem cells from bone marrow could be used to reconstitute the blood and immune system in patients with leukemia and other blood disorders. Their work led to the first successful human transplants in the 1960s. Initially, bone marrow transplants were highly experimental and confined to specialized centers. Over time, through continuous optimization of conditioning regimens, donor matching (HLA typing), and graft-versus-host disease management, the procedure became more standardized and scalable. Today, it is an established treatment for thousands of patients worldwide, facilitated by international donor registries and improved cryopreservation techniques that allow for broader accessibility. This evolution underscores the importance of starting with a highly controlled, expert-driven development phase, followed by a strategic expansion model to make cell therapies practical and available on a large scale.

Ultimately, if we are serious about completing the “last mile” in beta cell replacement, we must acknowledge that it is not a simple continuation of our current trajectory. It demands a fundamental shift in strategy—one that prioritizes not just scientific and technical innovation but also scalability, accessibility, and economic feasibility. Only by adopting this perspective can we transform beta cell replacement from experimental success into a truly viable treatment for millions of people with T1D. How long do we need to wait for having an exogenous insulin-free world? In all honesty, we do not know, but I am sure that the generation of individual with type 1 diabetes who will be definitively cured by beta cel replacement is already born. In the meantime, maintaining the parallel with the moon mission, we must push forward with unwavering commitment, as President John F. Kennedy said: “…We choose to go to the Moon in this decade and do the other things, not because they are easy, but because they are hard; because that goal will serve to organize and measure the best of our energies and skills, because that challenge is one that we are willing to accept, one we are unwilling to postpone, and one we intend to win, and the others, too (Rice Stadium on September 12, 1962)” This same spirit should guide our efforts in making beta-cell replacement a reality for those who need it most.

## Data Availability

The original contributions presented in the study are included in the article/supplementary material, further inquiries can be directed to the corresponding author.

## Footnotes

1
https://www.fiercepharma.com/pharma/once-valued-10b-bluebird-bio-sells-private-equity-firms-29m

2
https://www.fda.gov/media/170457/download

3
https://www.breakthrought1d.org/project-act/
